# Dissipation of S-metolachlor in plant and soil and effect on enzymatic activities

**DOI:** 10.1007/s10661-017-6071-7

**Published:** 2017-06-27

**Authors:** Elżbieta Wołejko, Piotr Kaczyński, Bożena Łozowicka, Urszula Wydro, Andrzej Borusiewicz, Izabela Hrynko, Rafał Konecki, Krystyna Snarska, Dorota Dec, Paweł Malinowski

**Affiliations:** 1 Division of Sanitary Biology and Biotechnology, Białystok University of Technology, Wiejska 45 E, 15-351 Białystok, Poland; 2 Laboratory of Pesticide Residues, Plant Protection Institute - National Research Institute, Chelmońskiego 22, Białystok, Poland; 3 Higher School of Agribusiness in Łomża, Studencka 19, 18-402 Łomża, Poland; 4 Division of Agrifood and Forestry Engineering, Białystok University of Technology, Wiejska 45 E, 15-351 Białystok, Poland; 5 Division of Statistics and Medical Informatics, Medical University of Bialystok, Szpitalna 37, Białystok, Poland

**Keywords:** S-Metolachlor, Dissipation, Soil, Maize, Acid phosphatase, Alkaline phosphatase, Dehydrogenase

## Abstract

The present study aimed at evaluating the dissipation of S-metolachlor (S-MET) at three doses in maize growing on diverse physico-chemical properties of soil. The effect of herbicide on dehydrogenase (DHA) and acid phosphatase (ACP) activity was estimated. A modified QuEChERS method using LC-MS/MS has been developed. The limit of quantification (0.001 mg kg^−1^) and detection (0.0005 mg kg^−1^) were very low for soil and maize samples. The mean recoveries and RSDs for the six spiked levels (0.001–0.5 mg kg^−1^) were 91.3 and 5.8%. The biggest differences in concentration of S-MET in maize were observed between the 28th and 63rd days. The dissipation of S-MET in the alkaline soil was the slowest between the 2nd and 7th days, and in the acidic soil between the 5th and 11th days. DT_50_ of S-MET calculated according to the first-order kinetics model was 11.1–14.7 days (soil) and 9.6–13.9 days (maize). The enzymatic activity of soil was higher in the acidic environment. One observed the significant positive correlation of ACP with pH of soil and contents of potassium and magnesium and negative with contents of phosphorus and organic carbon. The results indicated that at harvest time, the residues of S-MET in maize were well below the safety limit for maize. The findings of this study will foster the research on main parameters influencing the dissipation in maize ecosystems.

## Introduction

Maize (*Zea mays* L.) has high adaptive abilities and produces high fresh weight yields. Due to its cultivation in monoculture, more and more frequently, it decreases its yields owing to weed infestation, which at the early stages of growth competes with maize for nutrients and water (Sun et al. 2013; Cao et al. 2008). To prevent it, one uses herbicides, which play a key role in the maize conservation programmes. Furthermore, residues in the soil may pose a risk to consumers, which is why their constant monitoring is recommended.

The behaviour of herbicides in soil is complex and depends on many factors, such as the type of crop, soil pH, organic matter content, temperature, irrigation and light (Kah and Brown 2006) as well as microbial activity of the soil. Moreover, the properties of the herbicide molecule, such as water solubility, the octanol-water partition coefficient and the dissociation constant, are some of the most important factors influencing the bioavailability of these molecules (Chirukuri and Atmakuru 2015). The information about these processes is important for the evaluation of the dissipation rate of herbicides which allows for selecting the substances which may pose a potential threat to the natural environment as well as human and animal health.

Among herbicides, a very important one is S-metolachlor (2-chloro-N-(2-ethyl-6-methylphenyl)-N-[(1S)-2-methoxy-1-methylethyl]aceta-mide) used for selective weed, with wider use in sorghum, maize, cotton, potato, soybean, peanut or sunflower (Tomlin 2003). It is a chloroacetanilide herbicide classified as an inhibitor of very-long-chain fatty acid (VLCFA) formation (Tanetani et al. 2011). In its commercial formulation, S-metolachlor contains 88% S-enantiomer and 12% R-enantiomer, but the only biological active ingredient is S-enantiomer (Dale et al. 2006). Different behaviours in the soil sorption and dissipation processes are observed for this herbicide, which is related to its different chemical properties. S-metolachlor has a relatively high solubility in water (480 mg L^−1^), and it is highly soluble in acetone, ethyl acetate, toluene and xylene (PPDB 2014). Furthermore, its toxicological and environmental profile is favourable for mammals, birds and insects (honeybees included), except that it can be extremely toxic to fish and aquatic species (Yaw-Jian et al. 1999).

Following their release into the environment, pesticides may have many different fates and understanding their behaviour becomes of major scientific interest. Moreover, the pesticide evolution is conditioned by the microbiological composition of soil, in particular by the activities of soil enzymes (Gevao et al. 2000). According to Hussain et al. (2009), first, pesticides may disturb metabolism or soil enzymatic activities, and second, important information on pesticide transformation in soils may be provided by enzymes (Cai et al. 2015; Kalam et al. 2004). Among soil enzymatic activities, hydrolases are the most commonly measured activities in soils as potential indicators of the soil state (Śliwińska-Wyrzychowska and Nadgórska-Socha 2011; Floch et al. 2011). Moreover, phosphatases play a key role in phosphorus cycle in the soil (Schneider et al. 2001). According to Riah et al. (2014), they are capable of catalysing hydrolysis of esters and anhydrides of phosphoric acid in the soil and they are correlated positively to phosphorus stress and plant growth. Nowadays, many efforts have been made to understand the influence of pesticides on soil enzyme activities, dehydrogenase, protease and phosphatases. However, there is still little information on the effect of the S-metolachlor mixture widely applied in cultivation to influence the enzymatic activities in the soil (Nasreen et al. 2012).

Many studies have focused on the analyses of the behaviour of the herbicide in the soil, but only of individual molecules. However, as discussed by Bonfleur et al. (2015), interactions of additivity, synergism and antagonism may be possible among different herbicide molecules in a mixture. Therefore, further studies are needed to assess the influence of mixtures on herbicide sorption under different climate and soil conditions.

The objectives of the present study were fourfold: (1) to establish a simple residue analysis method for S-metolachlor in soil and maize, (2) to discuss the kinetic model of field dissipation, (3) to compare the speed of dissipation of S-metolachlor applied separately at different doses or in a mixture with a second herbicide in maize and soil in acidic and alkaline soil environment and (4) to investigate the influence of this herbicide and the physico-chemical properties of soil on enzymatic activities of soil.

The research results will contribute to the comprehension of the most important parameters responsible for the functioning of S-metolachlor in agriculture.

## Materials and methods

### Materials and reagents

Solvents (acetonitrile and methanol) were purchased from J.T.Baker (Deventer, The Netherlands). LC-MS-grade formic acid (98% purity) and 2,3,5-triphenyltetrazolium chloride (TTC) were obtained from Merck (Darmstadt, Germany), ammonium formate (>99%) from Fluka (Seelze-Hannover, Germany) and LC-grade water (18 MΩ cm) from a Milli-Q water purification system (Millipore Ltd., Bedford, MA, USA). QuEChERS Extract Kit was purchased from Agilent Technologies (Santa Clara, USA). Ammonium metavanadate, calcium lactate, sulphuric acid, hydrogen peroxide, nitric acid (68–70% *m*/*v*), hydrochloric acid (36.5–38% *m*/*v*) and chitin from shrimp shells (0.28–0.46 mm) were obtained from Sigma-Aldrich, Co.

The S-metolachlor standard and internal standard (isoprotron-d_6_) were obtained from Sigma-Aldrich (Steinheim, Germany). The solution of pesticide standard (around 500 μg mL^−1^) was obtained by dissolving reference standard in acetone. The working standard mixtures of 0.0005–1.0 μg mL^−1^ were prepared in methanol from the above stock standard by serial dilution. A solution of internal standard was prepared in methanol (0.5 μg mL^−1^). The stock, working standard solutions and internal standard were stored in a freezer at 4 °C until the analysis.

### Field trials

The field trials were designed according to GEP (Good Experimental Practice) in maize in randomized blocks in four repetition cycles. At each location, an area of 20 m^2^ (4 m × 5 m) was isolated in experimental plots for each combination in a given cycle. The experiments were conducted from May to July 2015 at two locations in north-eastern Poland in the Podlasie region (location 1 (L1)—Dobrzyniewo Duże 53° 12′ 40.0″ N 23° 02′ 28.8″ E, location 2 (L2)—Pawły 52° 56′ 22.2″ N 23° 18′ 22.2″ E).

The plots were selected on soil differentiated in terms of physico-chemical parameters of locations L1 and L2 (Table 1).

**Table 1:** Selected physico-chemical parameters of the soil samples

Soil samples	pH	mg/100 g			[%]	Granulometric structure of soil [%]			
		P_2_O_5_	K_2_O	Mg	C_org_	Sand 0.05–2.0 mm	Silt coarse 0.02–0.05 mm	Silt fine 0.002–0.02 mm	Clay < 0.002 mm
L1	7.4	19.3	18.9	7.6	0.8	77.10	9.41	11.29	2.20
L2	4.5	23.5	14.0	4.0	1.2	68.21	8.81	19.15	3.83

None of the plots had been treated with S-metolachlor in the past. Control samples were cultivated on a separate plot without pesticide treatment. Chemical procedures were performed with the use of various combinations of S-metolachlor in three doses of 863, 1200 and 1536 g ha^−1^ (S-m863, S-m1200 and S-m1536, respectively) and with another pesticide in two doses of 1200 and 1536 g ha^−1^ (S-m1200_MIX_ and S-m1536_MIX_, respectively) applied by means of a backpack compressed air sprayer (XR TeeJet 110 03 XR) at a spray rate of 200 L ha^−1^.

Soil and maize samples were collected about 2 h after the treatment as well as on the 1st, 2nd, 5th, 7th, 9th, 11th, 21st, 28th, 63rd and 85th days. The soil (1 kg) was randomly sampled in a depth of 0–20 cm in each plot using a sample drill at different spots. The maize samples (2 kg) were also collected randomly from each plot. Each maize sample was crushed thoroughly using the Waring Blender. The soil samples were air-dried and sifted through a 40-mesh sieve. Both maize and soil samples were stored at −20 °C until the analysis. All application doses of S-metolachlor were recommended by the producer. The treatments were carried out when maize was at the three-leaf stage (BBCH 13 for L1, 2015-05-28, and L2, 2015-05-29).

During the entire experimental period, rainfall was low and amounted to 6.4 mm. The average monthly air temperature was equal to 16.1 °C.

### Sample preparation and LC-MS/MS conditions

The plants were homogenized, and a part of each sample (10 g maize or soil) was put into a tube (50 mL). Next, 100 μL isoproutron-d6 (0.5 μg mL) (ISS, internal standard solution) and formic acid in acetonitrile (10 mL of 1%) were added. The whole mixture was quickly shaken (1 min), and a mix of salts (4 g anhydrous MgSO_4_, 1 g NaCl, 1 g trisodium citrate dihydrate and 0.5 g disodium hydrogen citrate sesquihydrate) and sorbent of chitin (1 g) were added. The flasks were vigorously shaken (1 min) and then centrifuged (10 min at 4000 rpm). Eight millilitres of the final extract was filtered (0.2 μm hydrophilic PTFE filter), and LC-MS/MS analysis was performed. A liquid chromatography system (Eksigent Ultra LC 100, Eksigent Technologies, Dublin, CA, USA) was operated at a flow rate of 0.3 mL min without split. Ten microlitres of extract was injected on an analytical column (Kinetex XB-C18, 1.7 μm, 2.1 × 50 mm, Phenomenex) maintained at 50 °C. The mobile phase comprised two stages: phase A (formic acid (0.5%), ammonium formate (2 mM) and methanol with formic acid (0.5%)) and phase B (ammonium formate (2 mM)). Ninety-five percent A and 5% B were held (0.5 min), increasing linearly to 5% A and 95% B (5.5 min), held (3 min) and returned to the initial composition (1 min). For mass spectrometric analysis, one used the system MS/MS (6500 QTRAP AB, SCIEX Instruments, Foster City, CA) equipped with an electrospray ionization source (ESI). To obtain a positive ion mode, one used nitrogen as the nebulizer gas, auxiliary gas and curtain gas at a pressure of 60, 50 and 30 psi, respectively. For identification of S-metolachlor, two multiple reaction monitoring modes (MRM) were used (Mol et al. 2015). The MRM of the most intense (357 340) was selected for quantification and the second (357 228) for qualification. For these two transitions, the collision energy was 12 and 28 eV, respectively. The declustering potential, entrance potential and collision cell exit potential were 30, 10 and 12 V, respectively. The retention time was 3.65 min.

### Method of validation

The validation of the proposed method was conducted according to SANCO guidelines (Document no. SANCO/12571/2013, 2014) of the European Commission (EC), using representative matrices previously checked to be free of pesticides. Recovery, precision, linearity, limit of detection (LOD) and limit of quantification (LOQ) were examined.

The validation parameters, i.e. the accuracy and precision, were evaluated by examination recovery and expressed as mean recovery and relative standard deviation (% RSD), respectively. To a blank matrix of maize/soil, calibration standards were added to obtain concentrations of 0.001, 0.005, 0.01, 0.05, 0.1 and 0.5 mg kg^−1^. The analysis was carried out in five replicates. Repeatability was observed for 5 days of the experiment. For each of the three different concentrations, five replicates were performed. Acceptable recovery should be in the range 70–120% with RSD <20%.

Linearity was assessed by the determination coefficient *(R* ^*2*^) of a matrix-matched calibration curve that was created based on six different concentrations of S-metolachlor.

The sensitivity was expressed by the limit of detection (LOD) and the limit of quantification (LOQ). The signal-to-noise ratio (S/N) criteria (LOD = 3 S/N, LOQ = 10 S/N) were applied.

Uncertainty measurement was estimated on the basis of the data obtained in the validation study. The expanded measurement of uncertainties was estimated by applying a “top-down” empirical model (coverage factor *k* = 2) at a 95% confidence level (Medina-Pastor et al. 2011).

### Assessment of enzyme activities

The dehydrogenase activity (DHA) was determined in the soil samples according to Casida et al. (1964), modified by Tabatabai (1994). All the samples contained 6 g soil and 1 mL of 3% aqueous solution of 2.3.5-triphenyltetrazolium chloride (TTC) and 2–4 mL of distilled water. The incubation time for all the samples was 20 h, and the incubation was conducted in the dark at 37 °C. After incubation, 20 mL methanol was added to all the samples and shaken vigorously and filtered. The DHA activity was measured at 485 nm. All results were expressed based on the dry weight of soil in micromoles of TPF per gram of soil per hour.

The extracellular soil enzymes (phosphomonoesterases: acid phosphatase (ACP) and alkaline phosphatase (ALP)) in the soil samples were assayed using the method of Tabatabai and Bremner (1969), modified by Dawson et al. (2007). Phosphomonoesterase activity has two pH optima of approximately pH 4–6 and 8–10. The phosphatase activities were determined by adding a modified universal buffer of suitable pH, 0.25 mL toluene and p-nitrophenyl phosphate solutions to the soil. The analysed samples were then incubated at 37 °C in a water bath for 1 h. After incubation, 1 mL 0.5 M CaCl_2_ and 4 mL 0.5 M NaOH were added, shaken vigorously and filtered. The released *p*-nitrophenol (*p-*NP) in micromoles of *p*-NP per gram of soil per hour was determined by absorption with a spectrophotometer at 410 nm.

The extinction of dehydrogenase and phosphomonoesterases was measured with the Perkin-Elmer Lambda 25 spectrophotometer.

### Statistical analysis

The comparison of several kinetic models of S-metolachlor dissipation can be evaluated to determine which model gives more complete information on the dissipation of pesticides. In this study, kinetic parameters were calculated for each concentration of herbicide and mixtures in the soil system. For the selection of the kinetic model that best describes the dissipation results, FOCUS work group guidance recommendations were followed (Document no. SANCO/10058/2005, version 2.0, 2006). First-order, biphasic, hockey stick and Gustafson and Holden models were fitted using available data. The first-order model was fitted using a generalized linear model based on the Gaussian family with logarithmic link. The Hockey stick model was calculated based on segmented adjustment to the first-order model, using segmented package Muggeo (2003). Biphasic and Gustafson and Holden models were fitted using the ordinary least square fit optimization procedure.

Goodness of fit was assessed using chi-square test based on standardized Pearson residuals, for each model. If the test *p* value was less than 0.05, the model was rejected due to poor fit. For model comparison, an Akaike (1974) information criterion (AIC) was calculated. The final model was selected using the AIC criterion from models that passed the goodness-of-fit test. Additionally, *R* ^2^ calculated as the complementary fraction of model deviance in null model deviance, with correction for multiplicity, was calculated. Given the selected model form, values for the time until 50 and 95% dissipation or, accordingly, DT_50_ and t_0.05_ values, were calculated.

The results of studies on activities of dehydrogenase, acid and alkaline phosphatase were subjected to analysis of variance to test the effect of various pesticides depending on the date and the place of sampling. Statistically significant differences were rated by Tukey’s test at a significance level at *p* < 0.05. The correlation between dehydrogenase, acid and alkaline phosphatase activity in soil and selected soil properties was calculated using Pearson’s correlation factor *r* for *p* ≤ 0.05. Calculations were performed using statistical environment R version 3.2.3 and Statistica 12.

## Results and discussion

Sample preparation and liquid chromatography-tandem mass spectrometry conditions were based on the method previously developed by Kaczyński et al. (2016) and adopted for this study. The validation study was performed using maize and soil samples previously analysed to be free of pesticides. The following parameters were evaluated: fortified recovery, the precision and the limits of detection (LOD) of the residue by means of the analytical method.

The S-metolachlor standard solution was fortified to the untreated soil maize at six concentration levels. The average recoveries for all concentrations were 92.7% (RSD = 6.3%) for soil and 89.3% (RSD = 5.3%) for maize, which was fully satisfactory. The LOQ (limit of quantification) was 0.001 mg kg^−1^, and the LOD (limit of detection) was 0.0005 mg kg^−1^ for soil and maize samples. Expanded measurement uncertainties were 18 and 23% for soil and maize, respectively.

### Dissipation of S-metolachlor under field conditions

**Fig. 1:**
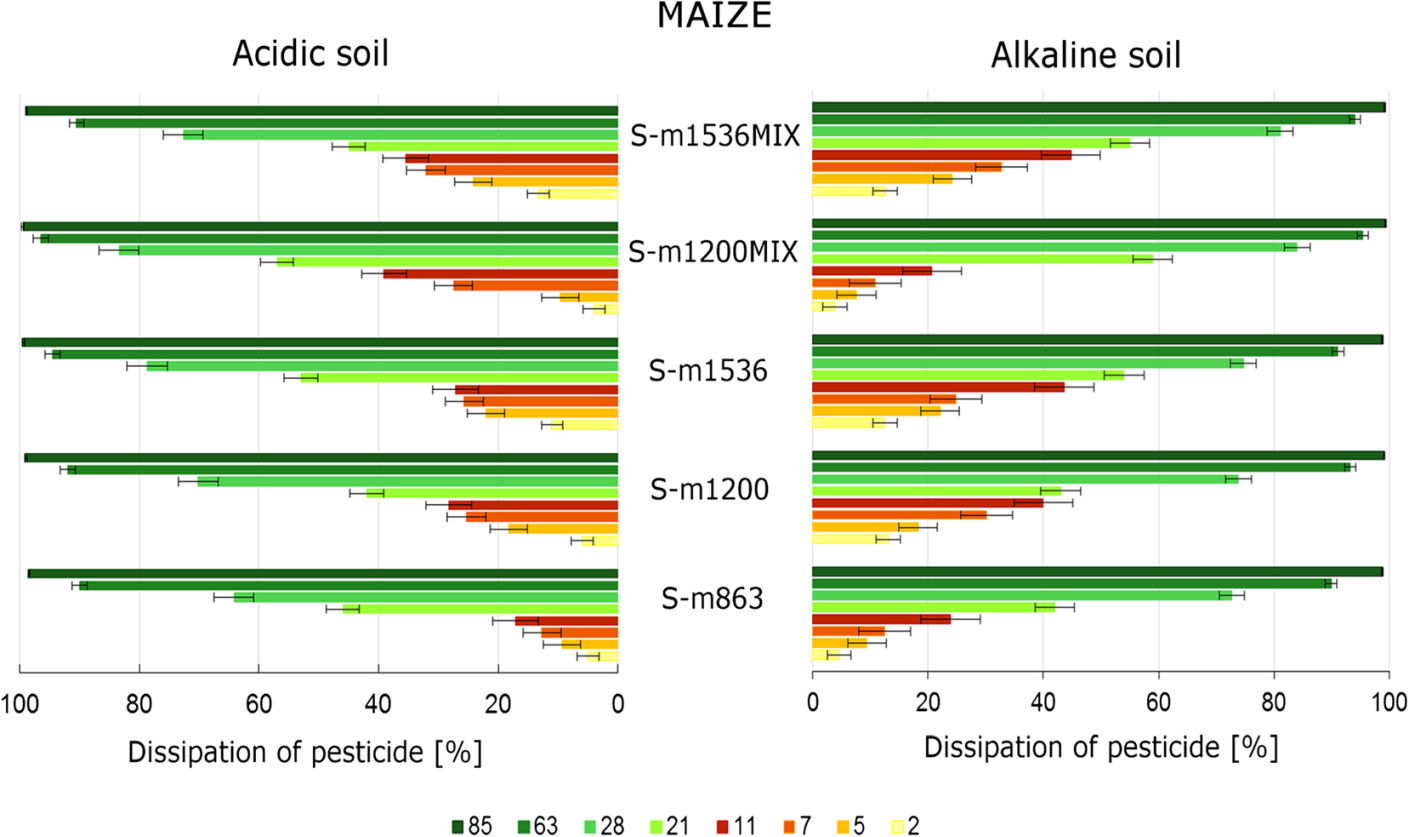
The changes of concentration of S-metolachlor applied separately and in mixture with another herbicide in maize

**Fig. 2:**
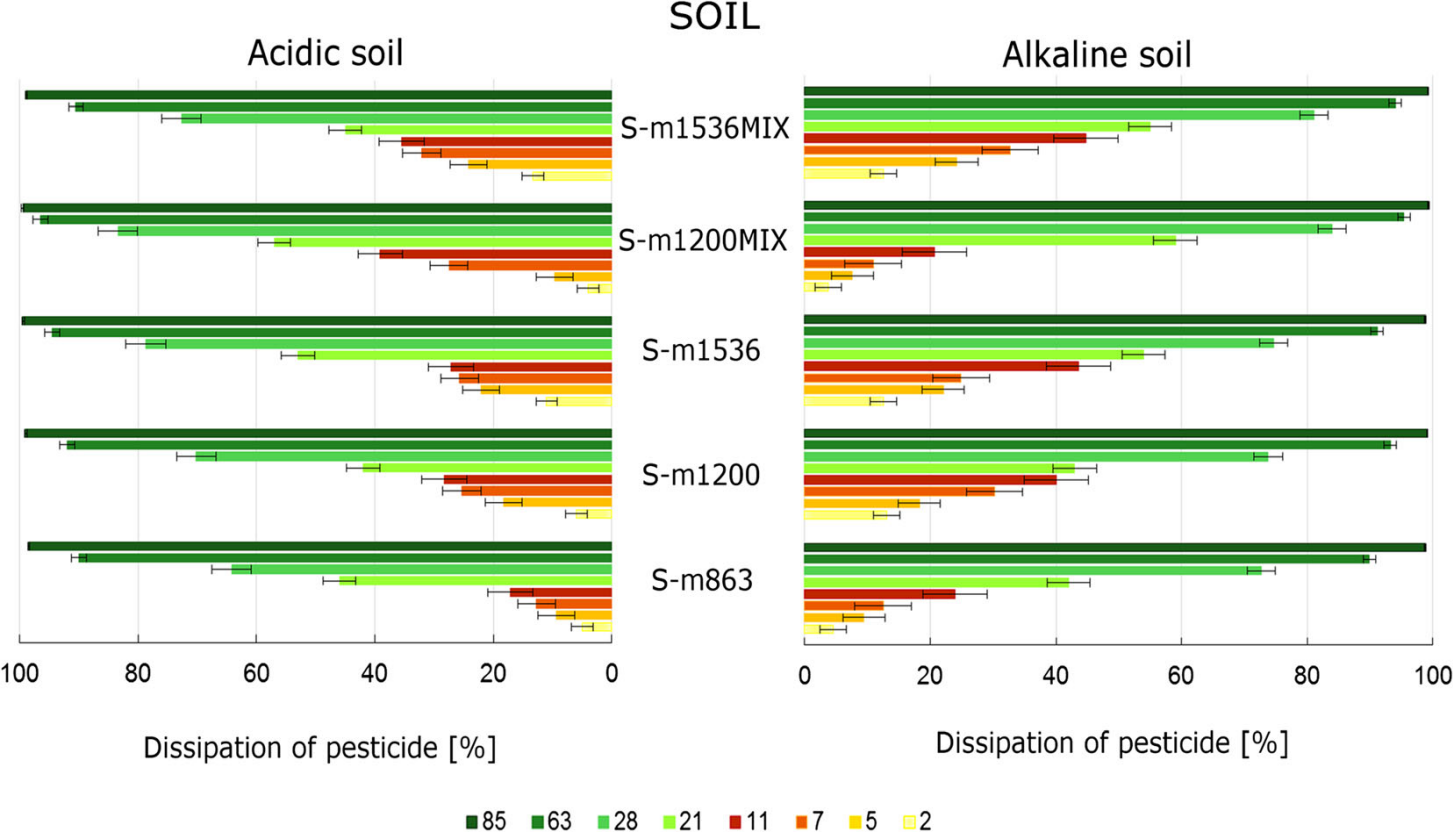
The changes of concentration of S-metolachlor after being applied separately and in mixture with another herbicide in soil

The changes of S-metolachlor concentration in the soil and maize were analysed, and the results are presented in Figs. 1 and 2 (contents as a percentage of the initially applied herbicide). The research was conducted on the maize crops in diversified soil in order to analyse granulometric structure, macronutrients, content of organic substances and pH of the examined soil. S-Metolachlor, as a single chemical substance and applied in mixture with another herbicide, is not the subject of the present study.

Table 2 presents the changes of S-metolachlor concentration in the soil and maize samples collected at the two locations as well as the percentage of its dissipation in Fig. 1. For the maize growing on the acidic soil, on the 2nd day after application of S-metolachlor, the percentage of its dissipation in maize was from 2 to 16%, whereas for the maize growing on the alkaline soil, the percentage value ranged from 11 to 25%. The dissipation of S-metolachlor in the alkaline soil was the slowest between the 2nd and 7th days, while that in the acidic soil was between the 5th and 11th days, and the dissipation of herbicide was approx. from 3 to 11% and from 1 to 12%, respectively.

**Table 2:** The concentration of S-metolachlor (in mg kg^−1^) in the soil and maize from two locations L1 and L2

Days	S-m863		S-m1200		S-m1536		S-m1200MIX		S-m1536MIX	
	L1	L2	L1	L2	L1	L2	L1	L2	L1	L2
Matrix										
Maize										
1	0.454	0.564	0.768	0.764	0.937	0.876	0.690	0.743	0.812	1.008
2	0.395	0.536	0.660	0.733	0.702	0.858	0.614	0.624	0.666	0.957
5	0.371	0.455	0.588	0.630	0.611	0.635	0.491	0.487	0.579	0.651
7	0.315	0.396	0.494	0.542	0.477	0.476	0.339	0.433	0.429	0.534
11	0.300	0.337	0.425	0.455	0.439	0.371	0.285	0.299	0.407	0.395
21	0.234	0.296	0.386	0.419	0.298	0.357	0.239	0.254	0.346	0.344
28	0.182	0.126	0.352	0.159	0.203	0.114	0.201	0.048	0.294	0.096
63	0.020	0.018	0.032	0.019	0.016	0.016	0.016	0.007	0.044	0.014
85	0.006	0.004	0.009	0.003	0.004	0.001	0.004	0.002	0.008	0.005
Soil										
1	0.702	0.864	1.053	1.200	1.138	1.536	1.110	1.210	1.239	1.536
2	0.670	0.821	0.915	1.128	0.996	1.367	1.068	1.161	1.083	1.332
5	0.637	0.783	0.860	0.980	0.886	1.196	1.025	1.117	0.939	1.164
7	0.614	0.754	0.734	0.896	0.855	1.141	0.989	0.877	0.834	1.043
11	0.534	0.716	0.632	0.860	0.641	1.118	0.880	0.737	0.683	0.991
21	0.407	0.467	0.600	0.696	0.523	0.722	0.455	0.520	0.557	0.845
28	0.191	0.309	0.276	0.358	0.288	0.328	0.177	0.200	0.234	0.419
63	0.071	0.087	0.072	0.097	0.101	0.085	0.051	0.042	0.075	0.147
85	0.009	0.013	0.009	0.012	0.014	0.009	0.008	0.005	0.010	0.017

*L1* alkaline soil, *L2* acidic soil

The biggest differences in the dynamics of S-metolachlor in maize were detected between the 28th and 63rd days, when the percentage of dissipation was above 85% for all doses applied. Moreover, one determined the highest ratio of differences in its concentration in the soil at both locations L1 and L2 between the 21st and 28th days, when the percentage of S-metolachlor dissipation differed by 30–60% on average. The biggest difference was observed for combinations S-m1200_MIX_ and S-m1536_MIX_ (Fig. 2, Table 2).

To some degree, soil parameters such as moisture, temperature and pH are subject to spatial and temporal variability; thereby, they may affect the determining persistence of the active substance of herbicides in the soil environment (Long et al. 2014). In our study, during the vegetation period, air temperatures were high and rainfall was low, which could influence the dissipation of S-metolachlor in soil and maize and extend its time distribution.

On the 1st day after application of the herbicide, the concentration of S-metolachlor in the soil from location L1 was within the range of 0.7–1.2 mg kg^−1^ and from location L2 within the range of 0.8–1.5 mg kg^−1^, while in maize, it was within the range respectively from 0.4 to 0.9 mg kg^−1^ and from 0.5 to 1.0 mg kg^−1^. After the 85th day from the application, the concentration of S-metolachlor in the soil and maize from location L1 was within the range from 0.006 to 0.01 mg kg^−1^ and that from location L2 was within the range from 0.004 to 0.01 mg kg^−1^ (Table 2). It indicates that both in plants and soil after the 85th day, there was almost complete dissipation of S-metolachlor.

### The kinetic model of dissipation of S-metolachlor

Parameters used for model selection are presented in Table 3. The hockey stick model for some data did not converge, due to lack of sufficient evidence of trend change. If this is the case, such models are marked by a minus sign in appropriate cells. For almost all analysed models, the chi-square test statistics was insignificant. Models with *p* value less than 0.05 were rejected; other models were retained for further analysis. After a close examination of the AIC values for each model, data indicates that the first order is the best model of describing the kinetics of S-metolachlor and mixture dissipations in the soil system.

**Table 3:** Parameters used for model selection

		Location	First-order			Biphasic			Hockey stick			Gustafson and Holden		
			AIC	χ^2^	*p* value	AIC	χ^2^	*p* value	AIC	χ^2^	*p* value	AIC	χ^2^	*p* value
Soil	S-m864	L1	−26.832	9.833	0.131	−20.832	8.067	0.152	−22.198	36.621	<0.001	−18.832	8.067	0.233
		L2	−26.511	8.907	0.178	−20.511	8.142	0.148	−22.892	11.059	0.025	−18.511	8.145	0.227
	S-m1200	L1	−17.999	9.897	0.129	−11.999	8.022	0.155	−16.267	47.191	<0.001	−9.999	8.023	0.236
		L2	−19.203	9.531	0.145	−13.203	8.073	0.152	−17.512	145.116	<0.001	−11.203	8.072	0.232
	S-m1536	L1	−24.794	9.913	0.128	−21.899	8.003	0.156	–	–	–	−17.058	8.005	0.237
		L2	−13.513	9.587	0.143	−7.513	8.053	0.153	−11.245	10.004	0.040	−5.513	8.053	0.23
	S-m1200_MIX_	L1	−12.876	9.679	0.138	−6.876	8.066	0.152	−10.672	7.966	0.092	−4.876	8.067	0.233
		L2	−19.270	9.651	0.140	−13.27	8.034	0.154	−17.030	7.984	0.092	−11.270	8.033	0.235
	S-m1536_MIX_	L1	−20.038	10.370	0.109	−16.808	8.007	0.155	−16.932	12.316	0.015	−12.239	8.006	0.237
		L2	−12.390	9.825	0.132	−6.395	8.009	0.155	–	–	–	−4.395	8.010	0.237
Maize	S-m864	L1	−38.436	9.280	0.158	−32.436	8.119	0.149	–	–	–	−30.436	8.119	0.229
		L2	−31.643	10.002	0.124	−25.643	8.034	0.154	−31.564	26.038	<0.001	−23.643	8.033	0.235
	S-m1200	L1	−21.020	9.501	0.147	−15.020	8.030	0.154	–	–	–	−13.020	8.030	0.235
		L2	−23.573	10.028	0.123	−17.573	8.041	0.153	−23.628	14.741	0.005	−15.573	8.041	0.235
	S-m1536	L1	−18.954	10.889	0.091	−17.339	8.094	0.151	–	–	–	−18.954	8.058	0.233
		L2	−18.214	9.761	0.135	−15.725	8.024	0.154	−16.237	11.003	0.026	−12.261	8.032	0.235
	S-m1200_MIX_	L1	−21.054	9.602	0.142	−20.347	8.084	0.151	−20.627	9.926	0.041	−18.410	8.049	0.234
		L2	−25.363	10.201	0.116	−22.498	8.013	0.155	–	–	–	−18.248	8.027	0.236
	S-m1536_MIX_	L1	−18.238	9.759	0.135	−16.815	8.101	0.150	−17.589	8.724	0.068	−11.916	8.035	0.235
		L2	−18.131	9.612	0.141	−17.402	8.017	0.155	−17.662	11.224	0.024	−13.631	8.039	0.235

*χ* ^*2*^ chi-square, *AIC* Akaike information criterion

**Fig. 3:**
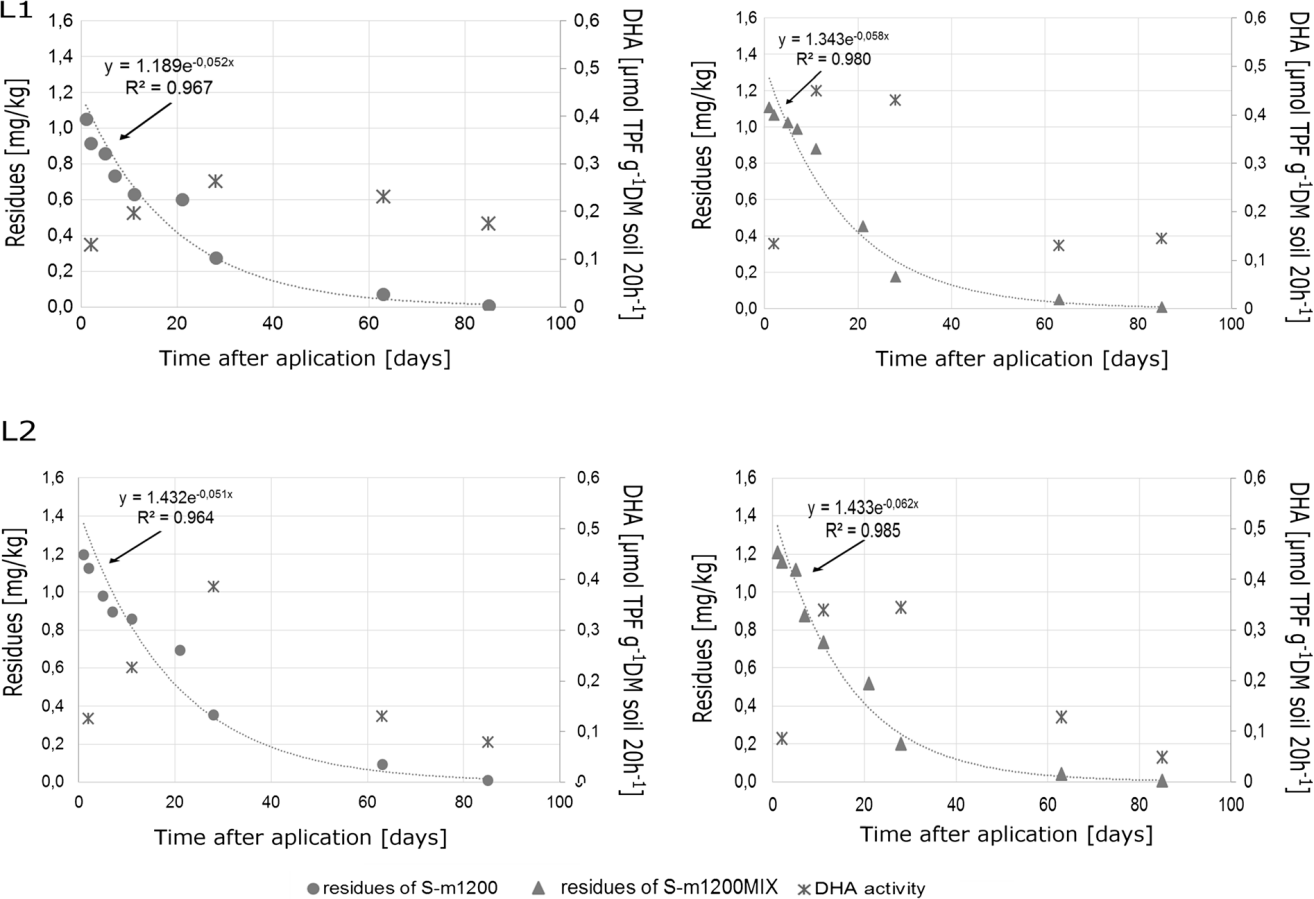
The dissipation kinetics of S-metolachlor in the dose of 1200 g ha^−1^ in soil and dehydrogenase activity in soil at the two locations (L1 and L2)

**Fig. 4:**
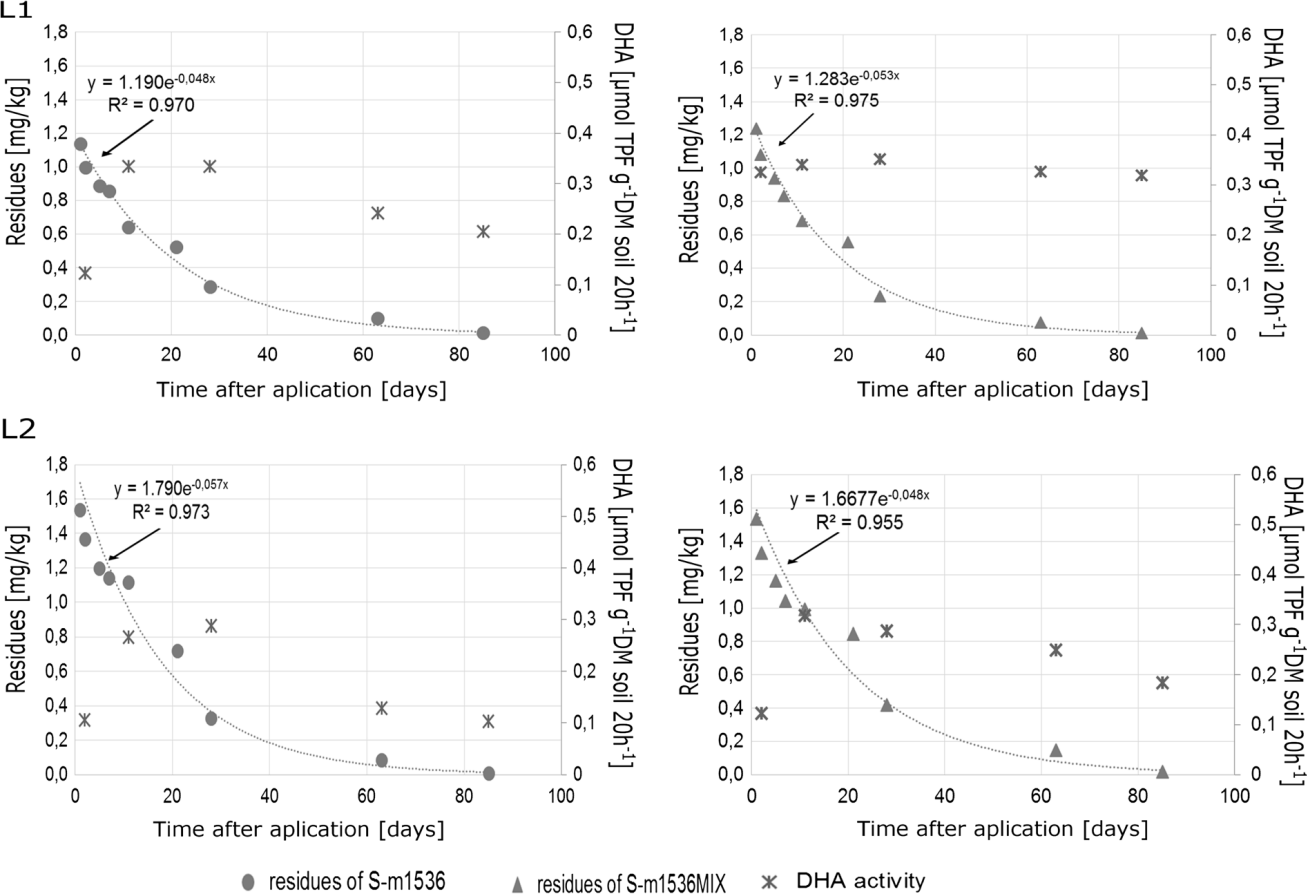
The dissipation kinetics of S-metolachlor in the dose of 1536 g ha^−1^ in soil and dehydrogenase activity in soil at the two locations (L1 and L2)

Table 4 and Figs. 3 and 4 present various values for final models. *R* ^2^ for each selected model was high (>0.90), further indicating good fit. The coefficients of determination *R* ^2^ for the first-order kinetic models were similar, since it ranged from 0.903 to 0.981 and from 0.955 to 0.985, respectively (Table 4).

**Table 4:** Model DT_50_ and t_0.05_ (in days) and statistical indices derived during modelling the kinetics for S-metolachlor dissipation in soil and maize

	Location	Regression equation	Correlation coefficient (*R* ^2^)	DT_50_	t_0.05_	Regression equation	Correlation coefficient (*R* ^2^)	DT_50_	t_0.05_
	Soil					Maize			
S-m864	L1	C = 0.857e^−0.0491t^	0.960	14.1	57.9	C = 0.515e^-0.052t^	0.984	13.9	44.8
	L2	C = 1.063e^-0.0470t^	0.970	14.7	65.1	C = 0.658e^-0.059t^	0.989	11.7	43.7
S-m1200	L1	C = 1.189e^−0.0523t^	0.967	13.3	58.3	C = 0.851e^-0.052t^	0.973	13.3	54.5
	L2	C = 1.432e^-0.0511t^	0.964	13.6	62.2	C = 0.936e^-0.064t^	0.986	10.8	45.8
S-m1536	L1	C = 1.190e^−0.0475t^	0.970	14.6	65.8	C = 0.909e^-0.063t^	0.993	11.0	46.0
	L2	C = 1.790e^−0.0567t^	0.973	12.2	51.2	C = 0.975e^-0.072t^	0.981	9.6	41.3
S-m1200_MIX_	L1	C = 1.343e^−0.0581t^	0.980	11.9	53.4	C = 0.679e^-0.058t^	0.985	12.0	44.9
	L2	C = 1.433e^−0.0622t^	0.985	11.1	60.4	C = 0.689e^-0.071t^	0.976	9.9	36.9
S-m1536_MIX_	L1	C = 1.283e^−0.0529t^	0.975	13.1	60.7	C = 0.810e^-0.051t^	0.972	13.9	54.6
	L2	C = 1.667e^−0.0481t^	0.955	14.4	71.2	C = 0.921e^-0.064t^	0.984	10.8	45.5

*DT*_*50*_ half-life value, *t*_*0.05*_ the degradation time up to the level of 0.05 value

The results of dissipation of S-metolachlor in doses 1200 and 1536 g ha^−1^ applied separately (S-m1200 and S-m1536) and in mixture with another herbicide (S-m120_MIX_ and S-m1536_MIX_) are presented in Figs. 3 and 4. The dissipation of a single pesticide in a lower dose of 1200 g ha^−1^ (S-m1200) in soil was described by the following equations: y = 1.18990e^−0.052x^ (L1), y = 1.4316e^−0.051x^ (L2). In turn, the dynamic of S-metolachlor in the same dose but in the mixture with another herbicide (S-m1200_MIX_) was described by the following equations: y = 1.3438e^−0.058x^ (L1), y = 1.4334e^−0.062x^ (L2). The dissipation dynamics of S-metolachlor in higher dose 1536 g ha^−1^, for S-m1536 and S-m1536_MIX_, in the soil samples could also be described as follows: y = 1.1901e^−0.048x^ (L1), y = 1.7907e^−0.057x^ (L2), and y = 1.2834e^−0.048x^ (L1), y = 1.6677e^−0.048x^ (L2), respectively. There was no difference in the decay rate of the substance, both applied as a single herbicide and in mixture with another. Moreover, the dose of herbicide did not influence dissipation dynamics.

Many researchers suggest that different kinetic models should be used to determine the best model of describing chemical substance decay in soil and plants. This is not easy because both soil and plants constitute a complex environment where the active substance is also degraded by microorganisms living there, which may affect its degradation, i.e. stimulate or inhibit it by passing through different metabolic pathways (Fantke and Juraske 2013; Sánchez et al. 2003).

Dissipation is a complicated process influenced by different physico-chemical and biological transformations, as stressed by Juhler et al. (2008), as a result of which the content of the active substance decreases over time. The half-life value DT_50_ is the time required for the dissipation process of a pesticide to be reduced to one half (EPA, U. S. E. P. and Fate, 2008). The DT_50_ value for the dissipation of S-metolachlor’s persistence in soil was expressed as a time after which 50% of the applied dose has been dissipated.

In the present study, the half-life was calculated for each concentration of S-metolachlor in soil and maize according to the formula DT50 = ln2*/*(−k). The half-life for the first-order kinetics was within the range of 11.1 to 14.7 days. The DT_50_ value for the dissipation of S-metolachlor in soil and maize for the first-order kinetics was within the same range as that reported by Caracciolo et al. (2005). In our study, the following values were reported: 11.1–14.7 days (soil) and 9.6–13.9 days (maize), while S-metolachlor half-lives in maize in Changchun and Beijing lasted longer: 6.68 and 4.84 respectively (Cao et al. 2008).

In the studies by Long et al. (2014), the DT_50_ value was within the range from 26.3 to 40.1 days for five different soils and it was higher than in the results of the present study. In turn, the estimated half-lives of S-metolachlor were shorter than those reported by Shaner et al. (2006), which lasted 18–27 days. The residue dissipation can be influenced by several parameters such as pesticide stability in soil and plants, pesticide application rate and frequency (initial concentration), weather (sun light, temperature, humidity and wind), microorganisms, pH of soil and water and nature of plant species (Lu et al. 2014; Fantke and Juraske 2013).

In order to obtain the concentration of 0.05 mg kg^−1^ (actual maximum residue level (MRL) for corn), the formula t_0.05_ = ln(0.05/C_0_)/(−k) was applied. Moreover, the t_0.05_ values obtained by interpolating between successive residue measurements were different and ranged from 51.2 to 71.2 days for soil, and 36.9 to 54.6 for maize. Comparing two locations, L1 and L2, the lowest t_0.05_ values in the soil were observed on the plot after the application of S-m1536 at location L1, and those in the maize on the plot after the application of S-m1200_MIX_ at location L2. Moreover, the highest values in the soil were observed at location L2 after the application of the pesticide S-m1536_MIX_, but in the maize—S-m1536_MIX_ at location L1. According to the first-order kinetic model (with the square of coefficient 0.955–0.993), S-metolachlor dissipated to concentration 0.05 mg kg^−1^ more rapidly in maize than in soil (Table 4).

### Influence of S-metolachlor of enzyme activities in soil

As it has been shown in the studies by Baćmaga et al. (2015), enzyme activities can reflect the changes in soil quality since they underpin nutrient cycling and function as indicators of the altered microbial community caused by environmental impact. Moreover, as suggested by Ushio et al. (2010), plant species as well as pesticide dissipation might influence the composition of microbial community and thus the extracellular activity of enzymes in soil.

**Fig. 5:**
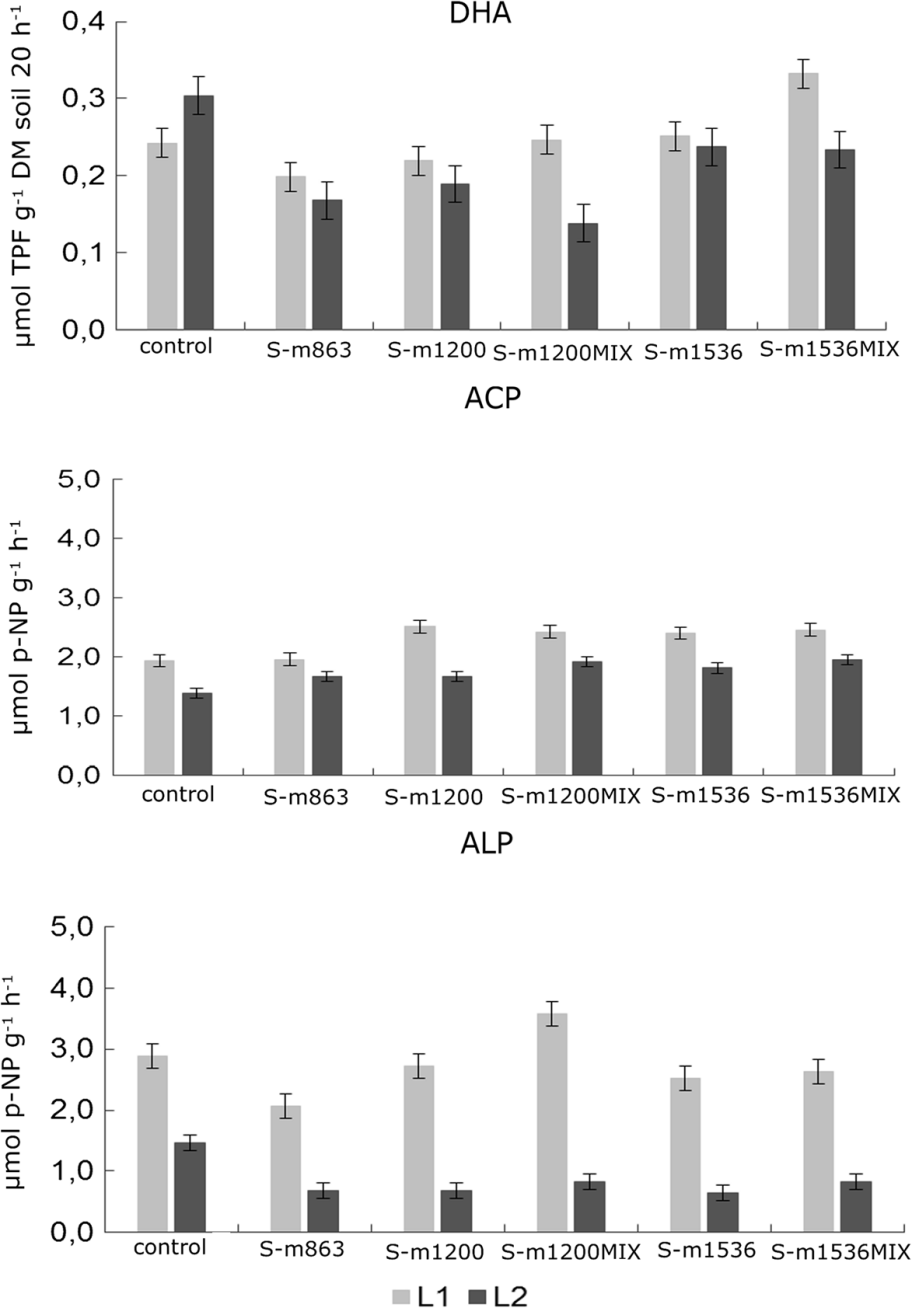
Average dehydrogenase activity and phosphomonoesterase in soil after application of S-metolachlor at both locations L1 and L2 (*bars marked with the same letters* indicate insignificant differences at *p* < 0.05; *error bars* represent the standard error) (*DHA* dehydrogenase activity, *ACP* acid phosphatase, *ALP* alkaline phosphatise)

**Fig. 6:**
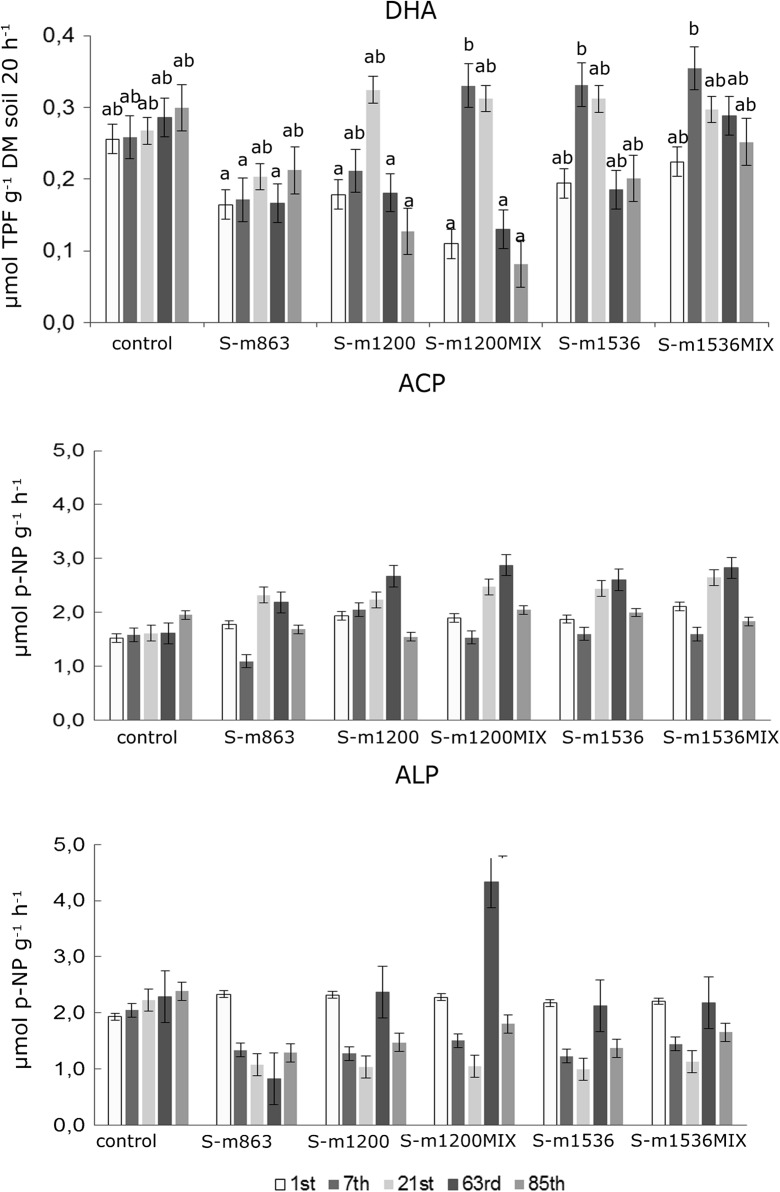
Average dehydrogenase activity and phosphomonoesterase in soil after application of S-metolachlor at various doses depending on the location (L1 and L2) of sampling (*bars marked with the same letters* indicate insignificant differences at *p* < 0.05; *error bars* represent the standard error) (*DHA* dehydrogenase activity, *ACP* acid phosphatase, *ALP* alkaline phosphatise)

The influence of S-metolachlor on dehydrogenase and phosphomonoesterase activity was investigated, and the results are presented in Figs. 5 and 6.

This study shows that the decomposition rate of S-metolachlor increased in time with the increase in the soil enzymatic activity (Fig. 5). The dehydrogenase activity was at the level from 0.17 μmol TPF g^−1^ DM soil 20 h^−1^ for S-m863 at L2 to 0.33 μmol TPF g^−1^ DM soil 20 h^−1^ for S-m1536_MIX_ at L1. At location L1, dehydrogenase activity was higher than at location L2.

The findings confirm that S-metolachlor application at a dose of 1200 g and 1536 g ha^−1^ (Fig. 6) may have contributed to the increase in dehydrogenase activity in the soil between the 2nd and 28th days after the application of the pesticide at both locations, i.e. L1 and L2. Moreover, one observed a decrease in dehydrogenase activity in the soil after the 28th day at the two locations, approx. from 12 to 70%. After the 85th day from the application of S-metolachlor, dehydrogenase activity in the soil was approx. 0.2 μmol TPF g^−1^ DM soil 20 h^−1^ for L1 and 0.1 μmol TPF g^−1^ DM soil 20 h^−1^ for L2.

It proves that the herbicide mixture or its metabolites may stimulate the activity of microorganisms in the 1st period, while the activity of DHA decreases with dissipation of the active substance. Furthermore, in the case of the application of the mixture of S-metolachlor and another herbicide (S-m1536_MIX_) (Fig. 5), DHA values were in the same range, i.e. approx. 0.33 μmol TPF g^−1^ DM soil 20 h^−1^ (L1) throughout the growing season. Bastida et al. (2008) report that physical and chemical indicators offer less information regarding the dynamics of soil than the biological ones, because the latter are closely related to the nutrient, particularly in terms of the influence of different uses and management practices of soil.

One of the findings of our study is that there were significant differences in dehydrogenase activity for the applied pesticides depending on the sampling time (Fig. 6). The minimum activity was 0.08 μmol TPF g^−1^ DM soil 20 h^−1^ for S-m1200_MIX_ 85 days after using S-metolachlor, while the maximum activity was 0.46 μmol TPF g^−1^ DM soil 20 h^−1^ for S-m1200_MIX_ 7 days after the pesticide application.

There were no significant differences in enzyme activity between the samples of the soil where pesticides were used and those from the control plots. The study shows that the average dehydrogenase activity on the control plots was equal to 0.27 μmol TPF g^−1^ DM soil 20 h^−1^, but after the application of S-metolachlor in doses of S-m863, S-m1200 and S-m1536, S-m1200_MIX_ and S-m1536_MIX_, it was lower than the activity on the control plots, on average by 10 to 30%. This indicates that using S-metolachlor separately and in mixture with another herbicide influenced negatively DHA activity, in particular, on acidic soils (L2). As noted in the studies by Nagatsuka and Furosaka (1980), the optimum range of pH for dehydrogenase activity is from around 7.4 to 8.9, which is confirmed by our own research at location L1. Moreover, the highest dehydrogenase activity was observed at location L1 at a dose of S-m1536_MIX_, where it increased by approx. 30% compared with the activity of DHA on the control plots (Fig. 5). According to Singh et al. (2003), increasing soil pH stimulates the activity of microorganisms, which, in turn, helps bacterial communities to adapt and develop specific gene enzyme systems for enhanced degradation of pesticides.

In the present study, there were no statistically significant differences in the activity of acid phosphatase (ACP) for the tested pesticides depending on the location and sampling time. The ACP activity ranged from 1.38 μmol *p*-NP g^−1^ h^−1^ (control samples taken at L2) to 2.52 μmol *p*-NP g^−1^ h^−1^ for the samples used in S-m1200 L1. Given the sampling period, the lowest activity of ACP was for S-m863–7 days after the application of the pesticide (1.10 μmol *p*-NP g^−1^ h^−1^), and the highest S-m1200_MIX_ was 63 days after the application of the pesticide. Generally, higher activity of acid phosphatase was observed for the tested mixture between the 21st and 63rd days after using S-metolachlor (Fig. 6).

In the present study, there was no impact of location and sampling time on the activity of alkaline phosphatase for individual pesticides. Its activity ranged from 0.65 μmol *p*-NP g^−1^ h^−1^ (for S-m1536 at L2) to 3.57 μmol *p*-NP g^−1^ h^−1^ (S-m1200_MIX_ at L1). A higher ALP activity was observed at L1 (2.07–3.57 μmol *p*-NP g^−1^ h^−1^) compared with L2 (0.65–1.46 μmol *p-*NP g^−1^ h^−1^) (Fig. 5). As for the date of sampling, ALP activity was the lowest for the S-m863 63 days after the application of the pesticide and it amounted to 0.83 μmol *p*-NP g^−1^ h^−1^, while the highest was recorded for S-m1200_MIX_ 63 days after the spray application—4.33 μmol *p*-NP g^−1^ h^−1^. The phosphomonoesterases (acid and alkaline phosphatases) play an important role in solubilizing insoluble phosphate monoesters (Richardson et al. 2000) and reflect soil biological health after pesticide application (Martinez-Salgado et al. 2010).

Nannipieri et al. (2011) report that the activity of enzymes in soil can also be significantly correlated with selected soil properties (e.g. pH, organic carbon). Table 5 shows Pearson’s correlation analysis of the content of dehydrogenase, acid and alkaline phosphatase activity in soil and selected soil properties. Acid and alkaline phosphomonoesterase activities were negatively correlated with contents of phosphorus (*r* = −0.45, *r* = −0.79, respectively), organic carbon (*r* = −0.50, *r* = −0.62, respectively), granulometric composition as silt fine (*r* = −0.47, *r* = −0.79, respectively) and clay (*r* = −0.34, *r* = −0.70, respectively) at *p* ≤ 0.05. As shown in the studies by Olander and Vitousek (2000), soil phosphatase activity is usually inversely related to soil phosphorus availability as a result of negative feedback by soil available phosphorus to the production and activity of phosphatase, which is confirmed by the present research. Furthermore, one observed a positive correlation between acid and alkaline phosphomonoesterase activities and contents of potassium (*r* = 0.43, *r* = 0.81, respectively), magnesium (*r* = 0.47, *r* = 0.78, respectively) and granulometric composition such as sand (*r* = 0.44, *r* = 0.79, respectively) and silt coarse (*r* = 0.38, *r* = 0.63, respectively). Moreover, dehydrogenase activity in soil was positively correlated only with silt coarse (*r* = 0. 34) at *p* ≤ 0.05 (Table 5).

**Table 5:** Correlation coefficients between the content of dehydrogenase, acid and alkaline phosphatase activity in soil and selected of soil properties

		DHA	ACP	ALP
	pH	0.26	0.48*	0.80*
mg/100 g	P_2_O_5_	−0.26	−0.45*	−0.79*
	K_2_O	0.27	0.43*	0.81*
	Mg	0.24	0.47*	0.78*
%	Corg	−0.22	−0.50*	−0.62*
Granulometric composition [%]	Sand 0.05–2.0 mm	0.27	0.44*	0.79*
	Silt coarse 0.02–0.05 mm	0.34*	0.38*	0.63*
	Silt fine 0.002–0.02 mm	−0.27	−0.47*	−0.79*
	Clay < 0.002 mm	−0.09	−0.34*	−0.70*

*DHA* dehydrogenase activity, *ACP* acid phosphatase, *ALP* alkaline phosphatase

*Correlation is significant at the 0.05 level

## Conclusions

1. On analysing S-metolachlor applied separately and in mixture with another herbicide, it can be concluded that the dissipation in both soil and maize was similar at the two locations, i.e. L1 and L2.
2. After a close examination of the *R* ^2^ values and half-life values DT_50_ for each fitting curve, the preliminary data indicates that the first-order kinetics is the best model of describing the kinetics of S-metolachlor and mixture dissipations in the soil system. This study may be helpful in setting MRL guidelines and safely using S-metalochlor separately and in mixture with another herbicide.
3. The dissipation of herbicide was faster in particular on alkaline soils compared with acidic soils. Yet, the soil type did not affect the dissipation of the herbicide in maize within the first 20 days; however, after the 21st day, one observed faster dissipation of the herbicide in plants growing on alkaline soils.
4. After the application of the pesticide, enzyme activity in the soil was not significantly different in comparison with the control plots. At various doses of pesticides, the enzymatic activity was higher in the soil samples taken at L1, which indicates that a higher pH of soil can positively influence the growth of enzymatic activity of soils and the dissipation of S-metolachlor.
5. Physical and chemical properties of soil influence microbial activity. There was a significant positive correlation between acid and alkaline phosphomonoesterase and soil pH and contents of potassium and magnesium, whereas there was a negative correlation of contents of phosphorus and organic carbon.

## References

[CR1] Akaike H (1974). A new look at the statistical model identification. IEEE Transactions on Automatic Control.

[CR2] Baćmaga M, Kucharski J, Wyszkowska J (2015). Microbial and enzymatic activity of soil contaminated with azoxystrobin. Environmental Monitoring and Assessment.

[CR3] Bastida F, Zsolnay A, Hernández T, García C (2008). Past, present and future of soil quality indices: a biological perspective. Geoderma.

[CR4] Bonfleur EJ, Tornisielo VL, Regitano JB, Lavorenti A (2015). The effects of glyphosate and atrazine mixture on soil microbial population and subsequent impacts on their fate in a tropical soil. Water Air & Soil Pollution.

[CR5] Cai Z, Li S, Zhang W, Ma J, Wang J, Cai J, Yang G (2015). Effects of the novel pyrimidynyloxybenzoic herbicide ZJ0273 on enzyme activities, microorganisms and its degradation in Chinese soils. Environmental Science and Pollution Research.

[CR6] Cao P, Wang X, Liu F, Zhao E, Han L (2008). Dissipation and residue of S-metolachlor in maize and soil. Bulletin of Environmental Contamination and Toxicology.

[CR7] Caracciolo AB, Giuliano G, Grenni P, Guzzella L, Pozzoni F, Bottoni P, Fava L, Crobe A, Orrù M, Funari E (2005). Degradation and leaching of the herbicides metolachlor and diuron: a case study in an area of Northern Italy. Environmental Pollution.

[CR8] Casida LE, Klein DA, Santoro T (1964). Soil dehydrogenase activity. Soil Science.

[CR9] Chirukuri R, Atmakuru R (2015). Sorption characteristics and persistence of herbicide bispyribac sodium in different global soils. Chemosphere.

[CR10] Dale LS, Galen B, David BW, Scott N (2006). Soil dissipation and biological activity of metolachlor and S-metolachlor in five soils. Pest Management Science.

[CR11] Dawson JJC, Godsiffe EJ, Thompson IP, Ralebitso-Senior TK, Killham KS, Paton GI (2007). Application of biological indicators to assess recovery of hydrocarbon impacted soils. Soil Biology & Biochemistry.

[CR12] Document no. SANCO/10058/2005, version 2.0. (2006), Guidance document on estimating persistence and degradation kinetics from environmental fate studies on pesticides in EU Registration, http://esdac.jrc.ec.europa.eu/public_path/projects_data/focus/dk/docs/finalreportFOCDegKinetics.pdf. Accessed 10 Apr 2017.

[CR13] Document no. SANCO/12571/2013 (2014), Guidance document on analytical quality control and validation procedures for pesticide residues analysis in food and feed. European Commission, http://www.eurlpesticides.eu. Accessed 13 Jan 2016.

[CR14] EPA, U. S. E. P A. Fate (2008), transport and transformation test guidelines OPPTS 835.6100 Terrestrial Field Dissipation. 712-C08-020.

[CR15] Fantke P, Juraske R (2013). Variability of pesticide dissipation half-lives in plants. Environmental Science and Technology.

[CR16] Floch C, Chevremont AC, Joanico K, Capowiez Y, Criquet S (2011). Indicators of pesticide contamination: soil enzyme compared to functional diversity of bacterial communities via Biolog Ecoplates. European Journal of Soil Biology.

[CR17] Gevao B, Semple KT, Jones KC (2000). Bound pesticide residues in soils: a review. Environmental Pollution.

[CR18] Hussain S, Siddique T, Saleem M, Arshad M, Khalid A (2009). Impact of pesticides on soil microbial diversity, enzymes, and biochemical reactions. Advances in Agronomy.

[CR19] Juhler RK, Henriksen TH, Ernstsen V (2008). Impact of basic soil parameters on pesticide disappearance investigated by multivariate partial least square regression and statistics. Journal of Environmental Quality.

[CR20] Kaczyński P, Łozowicka B, Hrynko I, Wołejko E (2016). Behaviour of mesotrione in maize and soil system and its influence on soil dehydrogenase activity. Science of the Total Environment.

[CR21] Kah M, Brown CD (2006). Adsorption of ionisable pesticides in soils. Reviews of Environment Contamination and Toxicology.

[CR22] Kalam A, Tah J, Mukherjee AK (2004). Pesticide effects on microbial population and soil enzyme activities during vermicomposting of agricultural waste. Journal of Environmental Biology.

[CR23] Long YH, Li RY, Wu XM (2014). Degradation of S-metolachlor in soil as affected by environmental factors. Journal of Soil Science and Plant Nutrition.

[CR24] Lu MX, Jiang WW, Jian Q, Shen Y, Liu XJ, Yu XY (2014). Persistence and dissipation of chlorpyrifos in *Brassica chinensis*, lettuce, celery, asparagus lettuce, eggplant, and pepper in a greenhouse. PloS One.

[CR25] Martinez-Salgado MM, Gutiérrez-Romero V, Jannsens M, Ortega-Blu R (2010). Biological soil quality indicators: a review. Mendez-Vilas A (ed) Current research, technology and education topics in applied microbiology and microbial biotechnology, 1.

[CR26] Medina-Pastor P, Valverde A, Pihlsotrm T, Masselter S, Gamon M, Mezcua M, Rodriguez-Torreblanca C, Fernandez-Alba AR (2011). Comparative study of the main top-down approaches for the estimation of measurement uncertainty in multiresidue analysis of pesticides in fruits and vegetables. Journal of Agricultural and Food Chemistry.

[CR27] Mol HGJ, Zomer P, Garcia-Lopez M, Fussell RJ, Scholten J, de Kok A, Wolheim A, Anastassiades M, Lozano A, Fernandez-Alba A (2015). Identification in residue analysis based on liquid chromatography with tandem mass spectrometry: experimental evidence to update performance criteria. Analytica Chimica Acta.

[CR28] Muggeo VMR (2003). Estimating regression models with unknown break-points. Statistics in Medicine.

[CR29] Nagatsuka T, Furosaka C (1980). Effect of oxygen tension on growth, respiration and types of bacteria isolated from soil suspensions. Soil Biology & Biochemistry.

[CR30] Nannipieri P, Giagnoni L, Landi L, Renella G, Bünemann E, Oberson A, Frossard E (2011). Role of phosphatase enzymes in soil. Phosphorus in action.

[CR31] Nasreen C, Jaffer Mohiddin G, Srinivasulu M, Rekha Padmini A, Ramanamma P, Rangaswamy V (2012). Interaction effects of insecticides on enzyme activities in black clay soil from groundnut (*Arachis hypogaea* L.) fields. Environmental Research, Engineering and Management.

[CR32] Olander LP, Vitousek PM (2000). Regulation of soil phosphatase and chitinase activity by N and P availability. Biogeochemistry.

[CR33] PPDB (2014). The FOOTPRINT pesticide properties database. UK: University of Hertfordshire; http://sitem.herts.ac.uk/aeru/footprint/es/index2.htm. Accessed 23 Jan 2016.

[CR34] Riah W, Laval K, Laroche-Ajzenberg E, Mougin C, Latour X, Trinsoutrot-Gattin I (2014). Effects of pesticides on soil enzymatic activities: general trends. Environmental Chemistry Letters.

[CR35] Richardson AE, Hadobas PA, Haynes JE (2000). Acid phosphomonoesterase and phytase activities of wheat (*Triticum aestivum* L.) roots and utilization of organic phosphorus substrates by seedling grown in sterile culture. Plant, Cell & Environment.

[CR36] Sánchez L, Peña A, Rasero SF, Romero E (2003). Methidathion degradation in a soil amended with biosolid and a cationic surfactant. Use of different kinetic models. Biology and Fertility of Soils.

[CR37] Schneider K, Turrion MB, Grierson PF, Gallardo JF (2001). Phosphatase activity, microbial phosphorus, and fine root growth in forest soils in the Sierra de Gata, western central Spain. Biology and Fertility of Soils.

[CR38] Shaner DL, Brunk G, Belles D, Westra P, Nissen S (2006). Soil dissipation and biological activity of metolachlor and S-metolachlor in five soils. Pest Management Science.

[CR39] Singh BK, Walker A, Morgan JAW, Wright DJ (2003). Effect of soil pH on the biodegradation of chlorpyrifos and isolation of a chlorpyrifos-degrading bacterium. Applied and Environmental Microbiology.

[CR40] Śliwińska-Wyrzychowska A, Nadgórska-Socha A (2011). The effect of heavy metal pollution and presence of *Lycopodium annotinum* on soil enzyme activity. Chemistry of Metals and Alloys.

[CR41] Sun Y, Xu Y, Sun Y, Qin X, Wang Q (2013). Dissipation and dynamics of mesotrione in maize and soil under field ecosystem. Bulletin of Environmental Contamination and Toxicology.

[CR42] Tabatabai MA, Weaver RW, Angle JR, Bottomley PS (1994). Soil enzymes. Methods of soil analysis: microbiological and biochemical properties. Part 2, SSSA Book Ser., 5.

[CR43] Tabatabai MA, Bremner JM (1969). Use of p-nitrophenyl phosphate for assay of soil phosphatase activity. Soil Biology & Biochemistry.

[CR44] Tanetani Y, Fujioka T, Kaku K, Shimizu T (2011). Studies on the inhibition of plant very-long-chain fatty acid elongase by a novel herbicide, pyroxasulfone. Pesticide Science.

[CR45] Tomlin CDS (2003). The pesticide manual.

[CR46] Ushio M, Kitayama K, Balser TC (2010). Tree species-mediated spatial patchiness of the composition of microbial community and physicochemical properties in the topsoils of a tropical montane forest. Soil Biology & Biochemistry.

[CR47] Yaw-Jian L, Karuppiah M, Shaw A, Gupta G (1999). Effect of simulated sunlight on atrazine and metolachlor toxicity of surface waters. Ecotoxicology and Environmental Safety.

